# Diversity begets diversity in mammal species and human cultures

**DOI:** 10.1038/s41598-020-76658-2

**Published:** 2020-11-12

**Authors:** Marcus J. Hamilton, Robert S. Walker, Christopher P. Kempes

**Affiliations:** 1 Department of Anthropology, University of Texas at San Antonio, San Antonio, TX USA; 2 Santa Fe Institute, Santa Fe, NM USA; 3 Department of Anthropology, University of Missouri, Columbia, MO USA

**Keywords:** Anthropology, Coevolution, Cultural evolution, Biodiversity, Biogeography, Macroecology

## Abstract

Across the planet the biogeographic distribution of human cultural diversity tends to correlate positively with biodiversity. In this paper we focus on the biogeographic distribution of mammal species and human cultural diversity. We show that not only are these forms of diversity similarly distributed in space, but they both scale superlinearly with environmental production. We develop theory that explains that as environmental productivity increases the ecological kinetics of diversity increases faster than expected because more complex environments are also more interactive. Using biogeographic databases of the global distributions of mammal species and human cultures we test a series of hypotheses derived from this theory and find support for each. For both mammals and cultures, we show that (1) both forms of diversity increase exponentially with ecological kinetics; (2) the kinetics of diversity is faster than the kinetics of productivity; (3) diversity scales superlinearly with environmental productivity; and (4) the kinetics of diversity is faster in increasingly productive environments. This biogeographic convergence is particularly striking because while the dynamics of biological and cultural evolution may be similar in principle the underlying mechanisms and time scales are very different. However, a common currency underlying all forms of diversity is ecological kinetics; the temperature-dependent fluxes of energy and biotic interactions that sustain all forms of life at all levels of organization. Diversity begets diversity in mammal species and human cultures because ecological kinetics drives superlinear scaling with environmental productivity.

## Introduction

The asymmetrical geographic distribution of life on Earth is often described by the latitudinal gradient of biodiversity^1–11^. Across many taxa, including mammals, birds, reptiles, amphibians^12,13^, plants^14–16^, marine bivalves^17^, ants^18^, liverworts^19^, and diseases^20^, species richness is highest near the equator and decays exponentially toward the poles. Moreover, these biodiversity gradients extend deep into the geological past^8,9,21,22^. It is now well-established that the biogeographic distribution of human cultural, linguistic, and economic diversity shows similar gradients^1,3,4,23,24^. While there is no unified mechanistic theory of biogeography to explain the origins of these biodiversity gradients, proposed hypotheses tend to focus on ecological (i.e., niche diversity), energetic (i.e., ecosystem kinetics), evolutionary (i.e., phylogenetic history), and/or historical mechanisms (i.e., dispersal)^12,14,25–29^. For cultural diversity similar mechanisms have been proposed, including latitudinal gradients in the length of the growing season^23,24^, environmental gradients^1,4,30^, and the timing of initial human colonization of particular continents^2^.

While latitudinal gradients may be empirically pervasive, latitude itself cannot be an explanatory variable as it is simply a geographic binning procedure that captures the steady loss biodiversity with increasing distance from the equator. Latitude indexes large-scale variation of Earth systems at many scales including the intensity of solar radiation, the length of the growing season, the distribution of precipitation, and the statistics of environmental temperature, all of which interact to constrain the distribution of environmental productivity around the planet^31^. Exactly how the productivity of local environments influences biodiversity has been central to ecological and evolutionary theory since Darwin and Wallace^32^.

Few studies have proposed mechanistic theory to explain why the global distributions of biodiversity and cultural diversity are so similar^3,4,6^. Ultimately, diversity must be correlated with the flux of energy through ecosystems as all organisms compete for environmental production to fuel metabolic processes of growth, maintenance and reproduction. Because environmental and climatic variation constrains the metabolic fluxes of free energy available to support biomass ecosystems differ in their capacity to support life^26,31^ and so the biogeographic distribution of biodiversity necessarily covaries with energy availability. The biogeographic distribution of human biomass is similarly constrained by geographic variation in energy availability despite technological innovations that enhance the ability of humans to compete for resources by reshaping their environments and redistributing materials, energy and information at multiple scales^33–37^.

To explain the ecological link between environmental production and species diversity the “more-individuals-hypothesis” proposed that increasingly productive environments support proportionally more individuals that belong to more species resulting in higher local diversity^25,38,39^. A similar hypothesis could be proposed for cultural diversity as human populations tend to be denser in more productive environments, which may lead to increased diversity. It is increasingly apparent, however, that species diversity is not simply a linear function of environmental productivity as the link between diversity and environmental productivity is more complex^26^. For example, while more productive environments house more species, tropical ecosystems house more diversity at all scales than would be expected by environmental production alone^15,26^. Similarly, studies of human cultures show that while diversity tends to be higher in more productive environments^1,3,4,6,23,24,40^ there are many other environmental, climatic, geological, and cultural processes that impact diversity at various scales^40–45^. For example, while global variation in hunter-gatherer population density, space use, and mobility is well-predicted by environmental productivity^44,46,47^, the spatial distribution of ethnolinguistic groups in general across the socioeconomic spectrum is better predicted by their level of sociopolitical complexity^45,48^.

In this paper, we use these studies as motivation to examine the macroecological relationship between mammal species diversity and human cultural diversity through the lens of the metabolic theory of ecology. Our goal in this paper is to explain why human cultural and mammalian diversity are so similarly distributed at the global scale and why both forms of diversity increase faster than would be predicted from environmental production alone. First, we derive predictive theory from the metabolic theory of ecology, which describes explicitly how the temperature-dependence of metabolic processes scales up to drive the temperature-dependence of species and cultural diversity. We then use this theory to develop a set of hypotheses that we test with global databases.

## Theory development

### Individual metabolism, temperature, and body size

We begin by considering the well-established link between individual and ecosystem level metabolism^49^. As first observed by Kleiber^50^, the basal metabolic rate of an organism, *B*, can be expressed in terms of body mass, *M*, as

1 $$ B = b_{0} M^{\alpha } $$

where *b*_0_ is a taxon-specific constant, independent of mass and temperature, and $\alpha \approx 3/4$ ( see^51–54^ for further discussions of the empirical form of Kleiber’s Law). The metabolic theory of ecology derives theory that explains how the scaling of mass and metabolism arises from the fractal-like, space-filling nature of optimized internal networks that distribute the energy and resources that sustain organisms, such as the vascular system^51,55–59^. The scale parameter, *b*_0_, is derived from the underlying biochemical kinetics of metabolism and, as such, depends on the temperature, *T*, at which an organism operates^57^. This is given by the exponential Arrhenius-Boltzmann factor, ${\text{exp}}\left( { - E_{B} /kT} \right)$, where *E*_*B*_ (~ 0.65 eV) is the average activation energy of the biochemical reactions contributing to metabolic processes of respiration, *k* is Boltzmann’s constant (8.62 × 10^–5^ eV K^−1^), and *T* is the absolute temperature at which the organism operates (°K)^60^. For endotherms, *T* is the internal body temperature, and for ectotherms, *T* is the ambient environmental temperature. Incorporating this temperature dependence into Eq. (1) yields the expression

2 $$ B = b_{0} e^{{ - E_{B} /kT}} M^{\alpha } . $$

which is the central equation of metabolic theory of ecology^57,60,61^.

### Scaling up from body size to ecosystem metabolism

**Figure 1:**
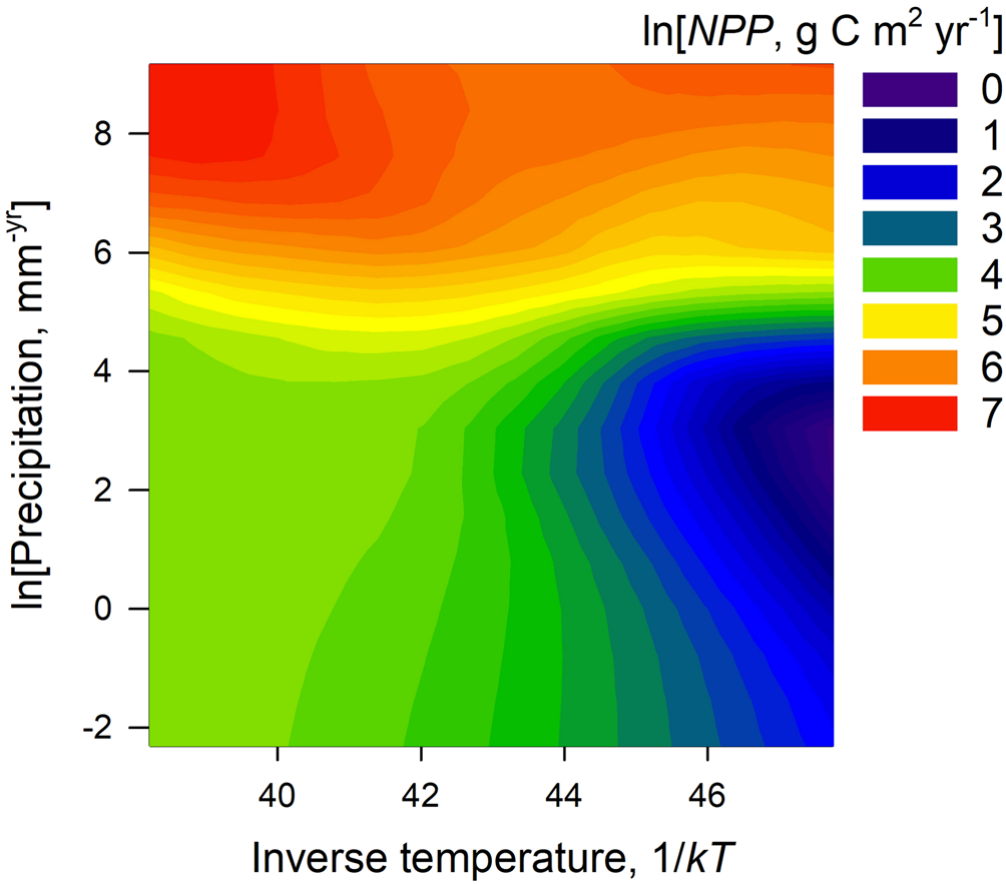
A 3D surface plot of net primary production (*z*) as a function of inverse temperature (*x*) and precipitation (*y*) in our data. The surface is a LOESS fit.

As ecosystems are composed of individual organisms, it follows that the respiration of an entire ecosystem is the sum over all individuals, the majority of which are microbes and plants^49^. Temperature is one of the primary drivers of energy flux in ecosystems^62^ the largest of which is gross primary production, *GPP*, the total mass of carbon entering an ecosystem through the conversion of solar radiation into biomass by photosynthesis over a given period of time^63,64^. At steady state, about half of *GPP* is respired by plants to support production, maintenance, and ion uptake^65^. The remainder is used by plants to fuel growth, and this is terrestrial net primary production (*NPP*), the net carbon gain in an ecosystem over a given period of time measured in units of g C m^−2^ yr^−1^. Following mass-energy equivalence, *NPP* is the total amount of ecosystem energy available for work (i.e., Gibbs free energy). It then follows that the flux of free energy in ecosystems is captured by the Arrhenius-Boltzmann temperature-dependence $NPP \propto e^{{ - E_{NPP} /kT}}$^49,66^. If $E_{NPP}$ is assumed to be the activation energy of photosynthesis, then holding all other ecological and evolutionary processes constant $E_{NPP} \approx 0.3$ eV. However, all is not constant as the kinetics of *NPP* responds to other rate limiting constraints such as stoichiometry, climatic constraints, and water-availability^26,31,62,67^. Here we measure the temperature-dependence of *NPP* from data: introducing annual precipitation, *R* (mm yr^−1^), as a rate-limiting constraint on the kinetics of *NPP*, we have

3 $$ NPP = c_{1} R^{{\beta_{NPP} }} e^{{ - E_{NPP} /kT}} , $$

where $\beta_{NPP}$ is an exponent capturing the effect of precipitation on *NPP* and *c*_1_ is a normalization constant independent of precipitation or temperature. We fit a multiple regression model to the logarithmic-transform of Eq. (3) and present the results in Table 1. Results show global scale variation in *NPP* is well-predicted by temperature and precipitation ($R^{2} = 92\%$), where the kinetics have a slope $E_{NPP} = 0.52$ eV and precipitation is a sublinear constraint on *NPP*, ${ }\beta_{NPP} = 0.72$. Figure 1 illustrates these relationships in a 3D surface plot of *NPP* as a function of temperature and precipitation.

**Table 1:** Regression coefficients for the multiple regression model of *NPP* as a function of inverse temperature 1/*kT* and precipitation *R*.

Term	Coefficient	95% C.I	T-value	*p*-value
Intercept	22.55	28.75–24.35	11.18	< 0.001
*E*_*NPP*_	0.52	0.44–0.60	− 13.14	< 0.001
β_*NPP*_	0.72	0.60–0.83	11.08	< 0.001

Model statistics: *d.f.* = 58, *R*^2^ = 92%, *p* < 0.0001.

### The kinetics of ecosystem complexity and biodiversity

Although energy availability within ecosystems is ultimately determined by temperature-dependent fluxes of carbon, increasingly productive environments are also more structurally complex. That is to say, a polar desert is not simply an energy-poor tropical rainforest, and a tropical rainforest is not simply an energy rich grassland. Increasingly productive environments not only have greater energy flux per unit area but also house more species^26^. Evolutionary explanations suggest speciation and extinction dynamics vary consistently across environments affecting the standing stock of diversity, and data suggest the tropics are more taxonomically and genetically diverse as a result^68,69^. Niche-based explanations suggest that in productive environments resources are apportioned in increasingly different ways, thus increasing the number of species that may co-exist in an ecosystem, and phylogenetically-related species have a tendency to inhabit similar niches in similar environments^70–72^. Ecological speciation explanations suggest increasing interactions between species in more productive environments promote enhanced diversity through increased competition and constraints on dispersal. The metabolic theory of ecology suggests a more general kinetic explanation, not necessarily exclusive of the preceding theories; because the pace of life is driven by metabolic processes, all the ecological and evolutionary dynamics that constitute the structure of ecosystems are governed by biological kinetics where biotic interactions of all kinds occur faster when the environment is warmer^26,57^. Diversity increases both as a function of more energy and faster biotic interactions in more productive environments.

Because human societies are also necessarily embedded within ecosystems, the abundance, density, and the intraspecific diversity of the human species are similarly subject to ecological kinetics. Groups of human compete with each other for finite sources of space and energy, as well as with other species, and cultural diversity is the result of intraspecific group competition and other evolutionary processes playing out over multiple generations^73–75^. In this paper we use ethnolinguistic diversity as our measure of cultural diversity. The term ethnolinguistic refers to a spatially and culturally discrete population of language speakers, which may or may not share a common language with other groups around the planet, such as English speakers in the British Isles, Australia, or the Caribbean, or Diné speakers in Alaska or the American Southwest. In both of these cases, each language is composed of multiple ethnolinguistic populations.

We model the kinetics of diversity by considering that the number of species increases with environmental productivity, and as the number of species increases there are more interactions between those species. We write the kinetics of diversity as a function of both the kinetics of energy flux and all other biotic interactions:

4 $$ S = c_{2} e^{{ - E_{S} /kT}} \propto e^{{ - \left( {E_{NPP} + E_{I} } \right)/kT}} , $$

where $E_{S}$ is the overall kinetics of diversity and $E_{I}$ is the kinetics of ecological interactions. Here, the kinetics of diversity, $E_{S}$, will be faster than the kinetics of ecosystem energy flux, $E_{NPP}$, when the kinetics of biological interactions $E_{I} > 0$. From Eqs. (3) and (4) we can then express species diversity, *S*, as a function of *NPP* yielding

5 $$ S = c_{2} NPP^{{\beta_{S} }} $$

where $\beta_{S} = E_{S} /E_{NPP} = \left( {E_{NPP} + E_{I} } \right)/E_{NPP} = 1 + \delta_{S}$, and so $\delta_{S} = E_{I} /E_{NPP}$. Therefore, diversity will increase superlinearly ($\beta_{S} > 1$) with environmental productivity whenever the kinetics of diversity are faster than the kinetics of environmental productivity ($E_{S} > E_{NPP}$). If there are no interaction kinetics ($E_{I} = 0$), diversity will increase linearly with environmental productivity, and if interaction kinetics are negative ($E_{I} < 0$)—from competitive exclusion, for example—diversity will increase sublinearly.

### Four hypotheses

To establish whether this simple model of ecological kinetics captures variation in mammal and cultural diversity we use data on the global distribution of mammal species and ethnolinguistic populations to test four hypotheses using predictions derived from this model:

H_1_: *Diversity is a function of kinetics.* We first test the hypothesis that diversity increases exponentially with temperature, as predicted by ecological theory at a rate consistent with the kinetics of environmental metabolism.
H_2_: *Diversity kinetics is faster than productivity kinetics*. The kinetics of diversity, *E*_*s*_, should be faster than the kinetics of environmental productivity, $$E_{NPP}$$, as species interaction rates, $$E_{I}$$, are predicted to be both non-zero and increase with environmental productivity, $$ NPP$$.
H_3_: *Diversity is more than environmental productivity*. Diversity, *S*, should increase with environmental productivity, *P*, at a superlinear rate given by $\beta_{S} = E_{S} /E_{NPP} > 1$.
H_4_: *Diversity kinetics are faster in more productive environments.* Holding *NPP* constant, diversity should increase with temperature, i.e., for a given range of *NPP* relatively warmer environments will be more diverse, and the slope of this response should increase in more productive environments.

## Results

### Hypotheses 1 and 2: Latitude and exponential temperature gradients

**Figure 2:**
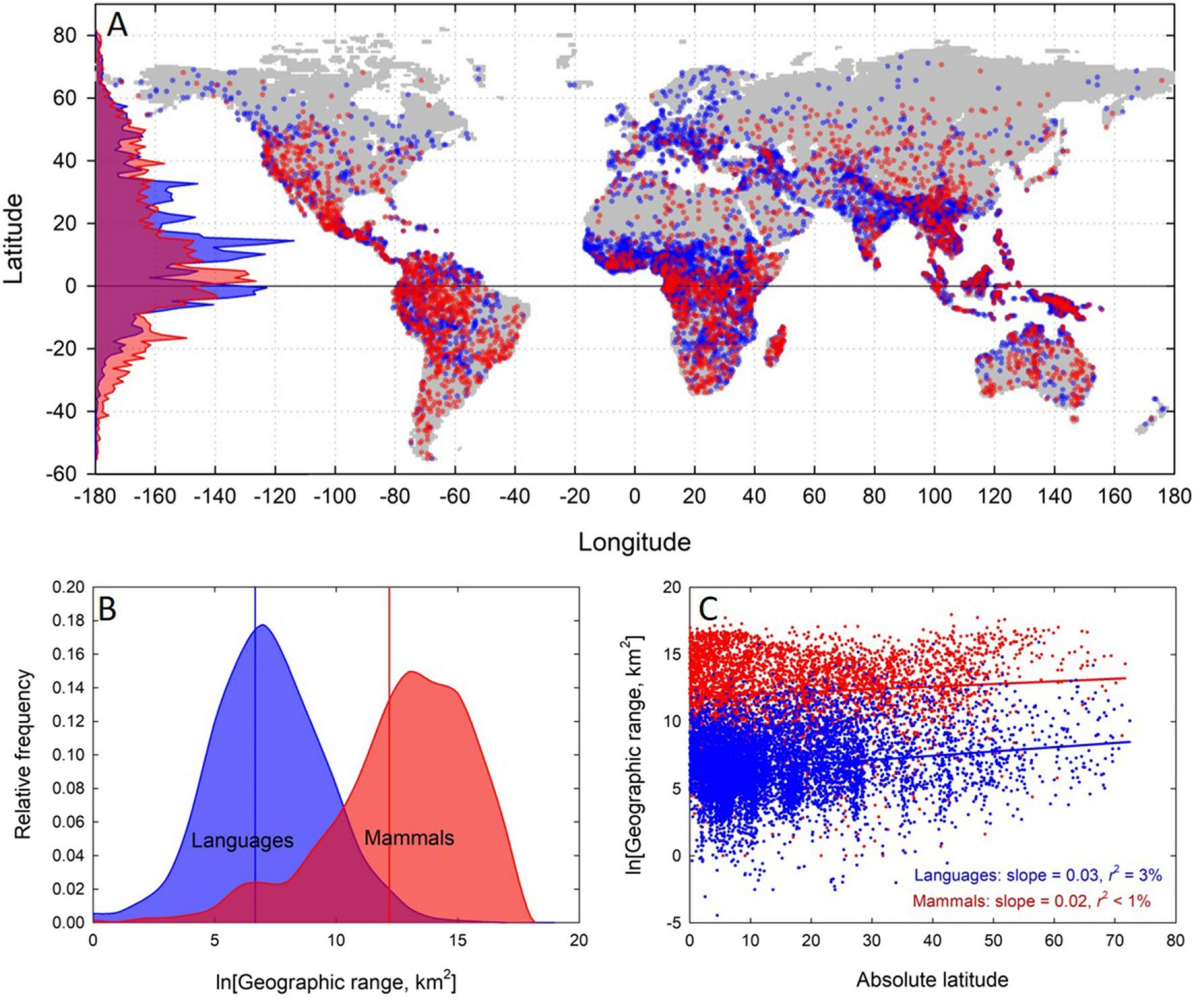
The biogeographic distribution of mammal species and human cultural diversity. (**A**) A map of the global distribution of human ethnolinguistic groups (blue) and mammal species (red). Each data point is the centroid of the geographic range of a species or a language (see “Methods” for further discussion). The underlying map is composed of 56,598 cells (0.5*0.5-degree latitude grids) comprising the global sampling procedure. Inset along the *y*-axis are distributions of relative diversity controlling for available landmass per 1-degree latitudinal bin (i.e., centroid density). Both forms of diversity are highest near the equator and decay toward the poles. The Figure was generated in Sigmaplot v. 13 (www.systatsoftware.com). (**B**) Relative frequency distributions of geographic ranges for languages and mammals, where the solid vertical lines are the means. The geographic ranges of mammals are on average about 250-times larger than those of languages. (**C**) A plot of geographic range of as a function of absolute latitude for both forms of diversity shows no systematic relationship indicating that the centroids of geographic ranges capture the biogeographic distribution. However, mammal geographic ranges are 250-times larger than ethnolinguistic ranges, as shown in (**B**).

Figure 2 shows that the spatial distribution of mammal species (red points) and languages (blue points) across the surface of the planet are similar. In Fig. 2A, each data point is the centroid of a geographic distribution. Inset along the Y-axis of Fig. 2A are distributions of the relative frequency of ethnolinguistic groups and the number of mammal species by latitude. Figure 2B shows that the average size of mammal species ranges is significantly greater than language ranges, but Fig. 2C show that geographic ranges do not vary systematically with absolute latitude. As such, the centroids of ranges shown in Fig. 2A accurately capture the biogeographic distribution of both forms of diversity around the planet.

**Figure 3:**
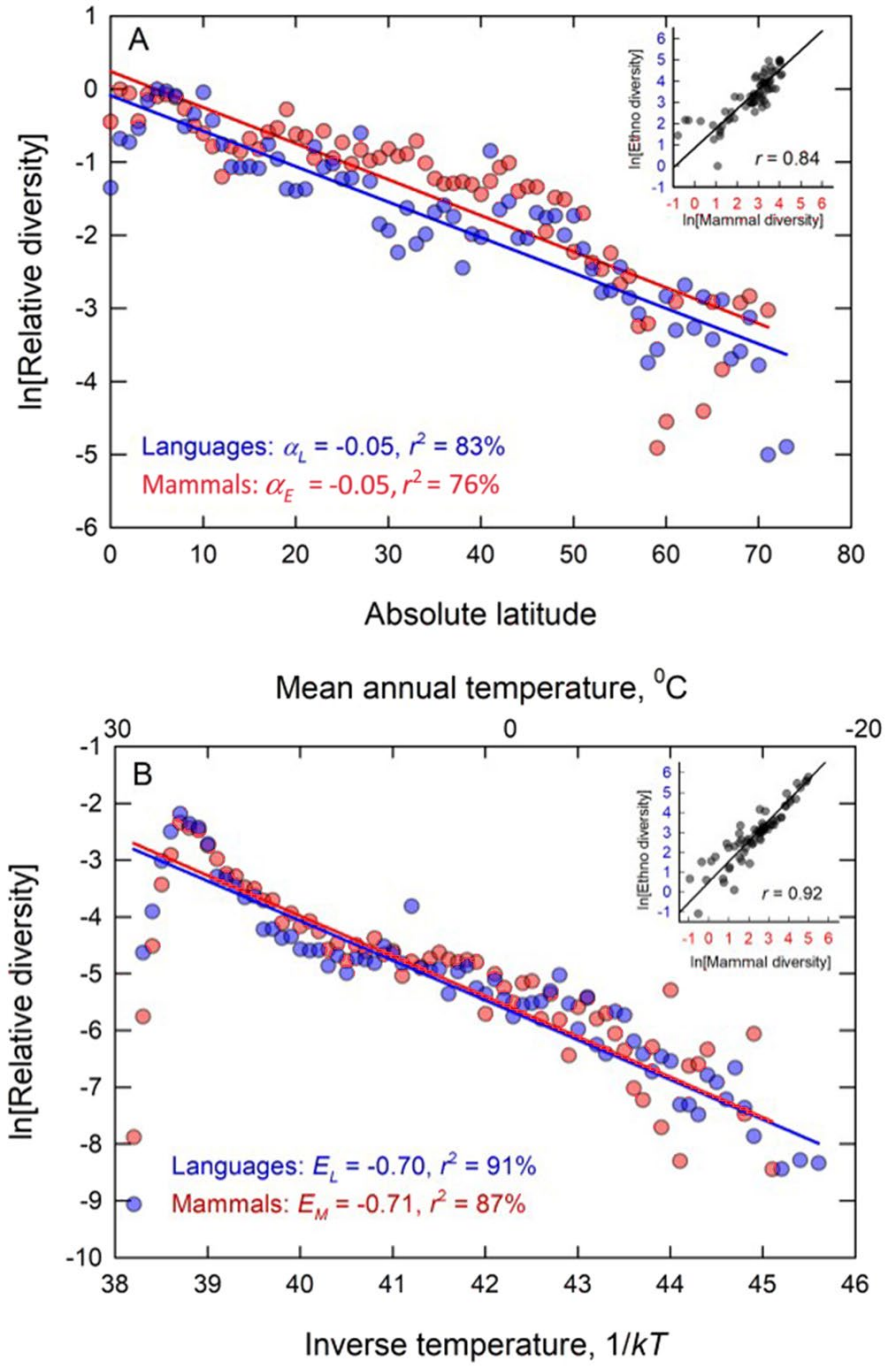
Latitudinal gradients (**A**) and temperature gradients (**B**) in relative ethnolinguistic diversity (blue points), and relative mammal species diversity (red points), corrected by available landmass (i.e., the area per 0.5*0.5 sampling bin). In both cases both forms of diversity decrease exponentially with latitude and temperature at statistically indistinguishable rates. Inset figures are the Pearson’s *r* correlations between the two forms of diversity. Solid colored lines are OLS regression fits.

Figure 3A shows that after controlling for available landmass cultural diversity and mammal diversity exhibit similar responses to absolute latitude. Here we plot relative diversity, $S^{\prime} = S_{i} /S_{{{\text{max}}}}$, for both mammal and cultural diversity and fit models of the form ${\text{ln}}S^{\prime} = {\text{ln}}S^{\prime}_{0} - \alpha L$, where $\alpha$ is the slope of the response and *L* is latitude. Results show cultural diversity decreases with absolute latitude at a rate $\alpha_{E} = 0.05 \pm 0.004$ (95% C.I.) (OLS regression: *d.f.* = 73, *r*^2^ = 83%, *p* < 0.0001) and mammalian diversity as $\alpha_{M} = 0.05 \pm 0.01$ (OLS regression: *d.f.* = 68, *r*^2^ = 76%, *p* < 0.0001); both forms of diversity decrease at a rate of ~ 5% per degree latitude, and the correlation coefficient between the two data sets is 0.84 (Fig. 3A inset).

In support of hypothesis 1, Fig. 3B shows the exponential relationship between relative diversity and environmental temperature (in °C (mean annual temperature) along the upper *x*-axis and inverse temperature 1/*kT* along the lower *x*-axis) for both forms of diversity. We fit regression models of the form $${\text{ln}}S^{\prime} = {\text{ln}}S^{\prime}_{0} + E/kT$$ is fit to both data sets. Cultural diversity decreases with temperature at a rate $$E_{L} = - 0.70$$ eV (0.65–0.76 95% C.I.) (OLS regression: *d*.*f*. = 65, *r*^2^ = 91%, *p* < 0.0001) and mammal species diversity decreases with temperature at a rate $$E_{M} = - 0.71 $$ eV (0.64–0.78) (OLS regression: *d*.*f*. = 60, *r*^2^ = 87%, *p* < 0.0001: for both data sets, statistical fits excluded the first three leftmost data points as they are outliers and have undue influence on the fit of the slope). The mammal and cultural slopes are not significantly different from each other and the two forms of diversity are highly correlated in space; $$r = 0.92$$ (Fig. 3B inset). As such, the latitudinal gradients of diversity in Fig. 3A are proxies of the temperature-dependent diversity shown in Fig. 3B. Importantly, the slopes of both the mammal and cultural fits are significantly steeper than the slope of NPP shown in Fig. 1 (*E*_*P*_ = _0.52_ eV), therefore providing support for hypothesis 2: The kinetics of diversity are faster than the kinetics of productivity.

### Hypothesis 3: Superlinear scaling of diversity and net primary production

**Figure 4:**
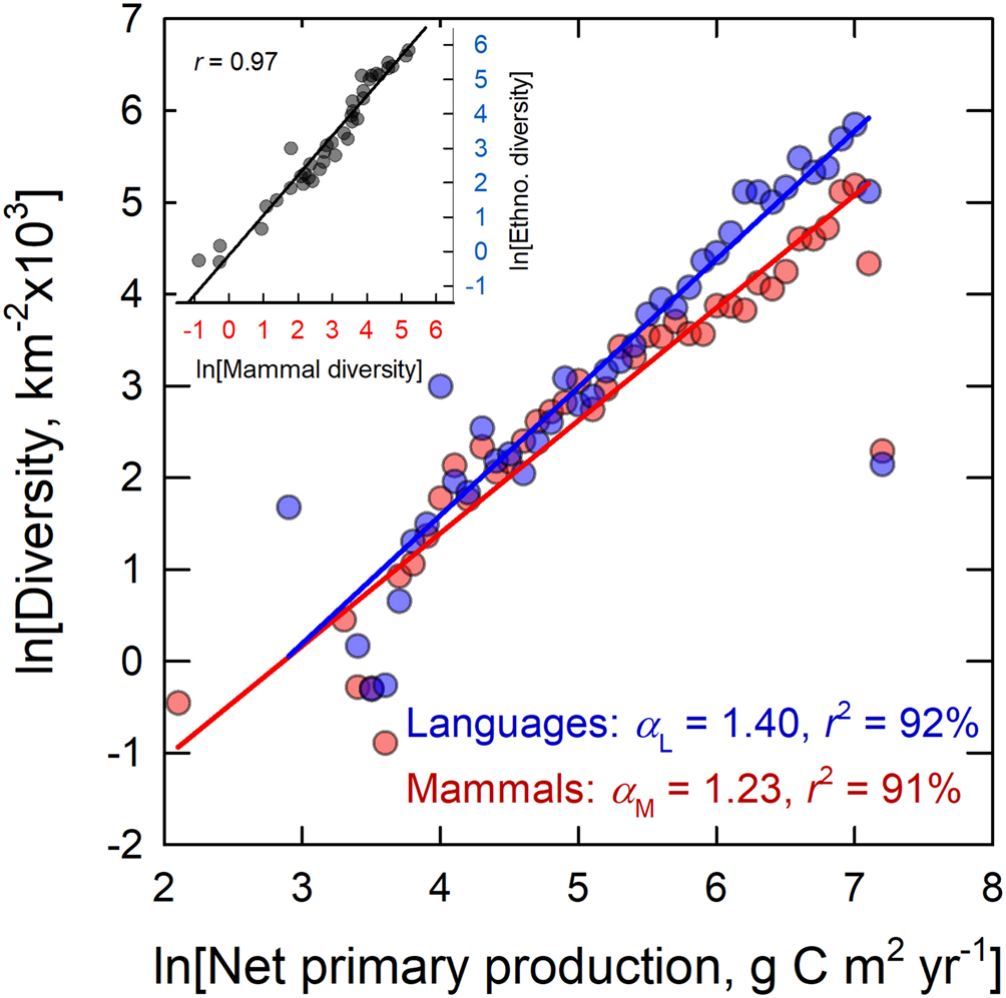
Ethnolinguistic and mammalian diversity per thousand square kms by net primary production. Inset figure is the Pearson’s *r* correlation of ethnolingistic and mammalian diversity. Solid colored lines are OLS regression fits; blue = ethnolinguistic and red = mammal.

Figure 4 shows that both forms of diversity scale superlinearly with net primary production, thus providing support for hypothesis 3. Cultural diversity increases superlinearly with *NPP* at a rate $\beta_{L} = 1.40$ (1.25–1.55) (OLS regression: *d*.*f*. = 40, *r*^2^ = 91%, *p* < 0.0001) and mammalian diversity increases as $\beta_{M} = 1.23$ (1.10–1.35) (OLS regression: *d*.*f*. = 36, *r*^2^ = 91%, *p* < 0.0001) (fits exclude the rightmost data point as it is an outlier and has a large influence of the fit of the slope). Both slopes are significantly greater than 1 and the data are highly correlated; $r = 0.97$ (inset figure). Moreover, the confidence intervals around the slopes both encompass their theoretically-predicted values, thus providing support for hypothesis 4. For mammals, the predicted slope based on the ratio of the kinetic exponents is $\lambda_{M} = E_{M} /E_{P} = 0.70/0.52 = 1.35$, which falls within the observed confidence interval 1.10–1.35. For cultural groups the predicted slope is $\lambda_{L} = E_{L} /E_{P} = 0.71/0.52 = 1.37$, which falls within the observed confidence interval 1.25–1.55.

### Hypothesis 4: Diversity kinetics and net primary production

**Figure 5:**
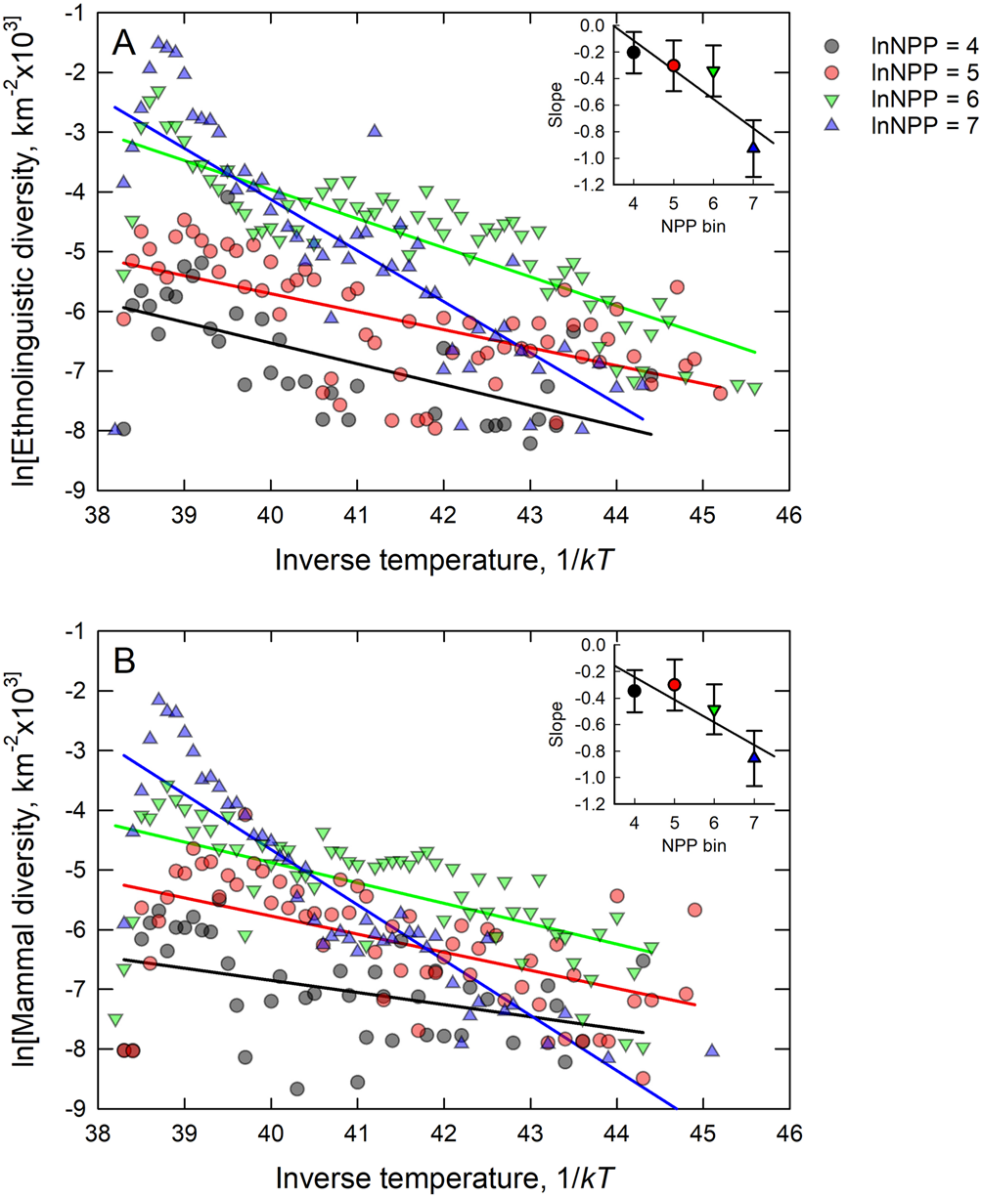
Ethnolinguistic (**A**) and mammal species (**B**) diversity by inverse temperature in bins of 1lnNPP. Colored lines are OLS regression fits. The inset figures plot the slopes of each bin against the ln*NPP* bin showing the general trend of faster kinetics with higher *NPP*. The data show the temperature dependent kinetics of diversity tend to be faster in more productive environments.

Figure 5A and B show that both forms of diversity increase exponentially with temperature at faster rates in more productive environments. Here the diversity data are aggregated into bins of width 1lnNPP from the 0.5*0.5-degree latitude sampling cells, and linear functions are fit to the data within each bin. Details of the regression models and the statistical results are given in the Supplementary Information. These figures demonstrate that for both cultural groups and mammal species diversity begets diversity as the interaction rates in more productive environments increase with increasing diversity, thus providing support for hypothesis 4.

## Discussion

Our results show that diversity begets diversity for both mammal species and cultural diversity because the kinetics of diversity are faster than the kinetics of environmental productivity resulting in superlinearity between diversity and environmental productivity. Increasingly productive environments are also more interactive meaning that not only is more energy available to support individuals, species, and communities, but there are increasingly different ways in which that energy is apportioned in the environment and thus competed over. More productive environments are also more complex, allowing more ways for diversity to be housed per unit area. To test this, we derived a simple model of diversity kinetics from well-established ecological theory from which we proposed four hypotheses whose predictions could be tested with data. Our results provide support for the four hypotheses posed in this paper, suggesting both mammal and ethnolinguistic diversity are positively density-dependent.

Our first hypothesis proposed that diversity is a function of kinetics. For both mammal species and cultural populations diversity increases with temperature exponentially at rates consistent with ecological kinetics (Fig. 3B)^62^. As such, a linear increase in temperature leads to a multiplicative increase in diversity and the rate of this increase is well-predicted by ecological theory indicating that the rate of increase in diversity is consistent with the overall kinetics of ecosystem metabolism. This means that for human cultures richer interactive environments are increasingly fragmentary and thus more culturally diverse and so increasing interaction results in more competition, not cooperation. Moreover, the kinetics of cultural diversity are mathematically and statistically similar to the kinetics of mammalian diversity, a process which happens across a much broader range of taxonomies, body sizes, life histories, and ecologies. Perhaps increasingly complex environments provide an increasingly diverse range of niches for humans to exploit economically by adopting a range of different lifestyles and technological innovations, such as foraging, farming, or horticulture. If so, the results shown here suggest that ecological kinetics constrain the availability of ecological niches for humans and mammals in similar ways.

Our second hypothesis predicted that kinetics of diversity is faster than the kinetics of environmental productivity. This is because the kinetics of diversity is predicted to be a combination of the kinetics of productivity and interaction. Results show that this prediction is supported by data where the kinetics of both mammal and cultural diversity ($E_{M} = 0.70$ and $E_{L} = 0.71$, respectively) are both significantly faster than the kinetics of *NPP*, ${ }E_{NPP} = 0.52$. Therefore, cooler less productive environments lose ethnolinguistic groups and mammalian species at a faster rate than would be predicted by the decrease in productivity alone. Thus, the change in diversity across environments is not only a function of energy availability, but niche complexity.

Our third hypothesis predicted the scaling of diversity and productivity to be superlinear. Data show that for both mammal species and human cultures diversity scales superlinearly with environmental productivity (for mammals $\beta_{M} = 1.23$ and for human cultures $\beta_{L} = 1.40$), and the scaling exponent is consistent with the predicted ratio of kinetic terms (Fig. 3B). This superlinearity describes increasing returns to scale in diversity with environmental productivity; for mammalian species a doubling of environmental productivity leads to ~ 23% more diversity than would be predicted by environmental energy availability alone, and in human cultures this increase is ~ 40%. The exact mechanisms that drive these increases are the subject of ongoing work.

Our fourth hypothesis predicted that as environmental productivity increases the temperature-dependence of the diversity response should also increase (Fig. 4). This result may be the strongest demonstration of the kinetic hypothesis as it shows holding environmental productivity constant, warmer environments are not only more diverse than less productive environments, but the rate of increase in diversity is faster in more productive environments. Richer environments house more species that interact with each other at increasingly faster rates. Therefore, mammalian and cultural diversity are both consistent with Red Queen dynamics driven by the temperature-dependence of interaction rates^76^.

This biogeographic convergence of biological and cultural diversity is striking given the differences in the evolutionary mechanisms and time scales of biological and cultural evolution^77,78^. Perhaps most obviously, by definition, mammal species diversity is interspecific whereas cultural diversity is intraspecific. Mammalian speciation occurs on time scales of millions of years while human languages speciate orders of magnitude faster at time scales of hundreds to thousands of years. Moreover, the pathways and currencies of biological and cultural evolution differ widely^79–81^, and so an a priori explanation of why these different evolutionary mechanisms should converge on similar biogeographic distributions is not obvious from the dynamics of the evolutionary mechanisms themselves.

However, it is interesting to note that while the centroid density of diversity is similar (Fig. 2A), the geographic ranges of mammal species and ethnolinguistic populations are quite different. Figure 2B and C show that on average mammalian geographic ranges are nearly 250-times larger than ethnolinguistic ranges. This suggests that while the centroids of their distributions converge, there are different dispersal mechanisms that impact the magnitude of their ranges. The larger geographic ranges of mammals could reflect the greater opportunities for species to co-occur in space across a broader range of body sizes and niches than is possible for humans. It may also be the case that faster rates of diversification in languages compared to species results in more restricted geographic ranges. As a result, the spatial turnover of mammalian and cultural diversity will differ at different spatial scales (i.e., differences in alpha, beta, and gamma-diversity^82^).

While biological and cultural evolutionary processes might be similar in principle^35,73,83,84^ and even share similar mathematical representations, the detailed mechanisms of the two evolutionary processes are entirely different. Biological evolution is a statistical consequence of the differential transmission of genetic and epigenetic information over generations in response to environmental selective constraints that impact the ability of individuals to compete for finite resources and produce viable offspring, and where speciation is the macroevolutionary consequence over large time scales^85,86^. Cultural evolution is a statistical consequence of the differential transmission of social information at many scales of social organization via multiple transmission pathways in both space and time that similarly impacts the ability of individuals to compete for finite resources and produce viable offspring^73,74,84^. Languages, cultures, and economies turnover at much faster time scales the biological species, from the near instantaneous to many thousands of years^77,87^. One axiomatic implication of the results shown here is that the gradients of diversity that emerge in response to ecological kinetics must be independent of specific evolutionary mechanism (i.e., genes vs. culture), otherwise they would be very differently distributed in space. It may be the case that large-scale geological, climatic, and environmental constraints impact speciation and extinction processes in both systems in similar ways across gradients of environmental productivity, despite the different evolutionary inheritance mechanisms and time scales involved. As such, the standing stock of diversity at any one location on the planet may be quite different between mammal species and cultures, but the gradients are similar.

Whatever the specific similarities or differences between biological and cultural evolution the general mechanism of convergence proposed here is kinetic. In general, more productive environments are warmer than less productive environments, and with increasing temperature not only is more energy available but there are increasing rates of interaction, including competition, specialization, and mutualisms, all of which combine to increase diversity above levels predicted by environmental productivity alone. Diversity increases superlinearly with environmental productivity because more productive environments are also more complex.

## Methods

### Temperature and environmental productivity data

We used the WorldClim database for mean annual temperature in °C and mean annual precipitation in mm yr^−1^ at 10 arc min resolution (version 1.4; https://www.worldclim.org/bioclim). WorldClim provides interpolated global climate data for stations with 10 to 30 years of data^88^. We used estimates of annual net primary productivity (g C m^−2^ yr^−1^) from an average of 17 global models at a spatial resolution of 0.5 degrees^89^. All data were first projected with the WGS 1984 Cylindrical Equal Area projection and then processed across our language polygons using the Zonal Statistics tool in ArcGIS. The Global Self-consistent, Hierarchical, High-resolution Shoreline dataset was used as the basemap^90^.

### Mammal and ethnolinguistic data

Ethnolinguistic data were downloaded from the Ethnologue, and centroids (in decimal degrees of latitude and longitude) were recorded for each individual language polygon (*N* = 7627). Terrestrial mammal data were downloaded from PanTHERIA and centroids (in decimal degrees of latitude and longitude) were recorded for each species (*N* = 5089). Average temperature, annual precipitation, and net primary productivity were the extracted for each ethnolinguistic population and mammal species. Data layers were compiled in ArcGIS and then analyzed statistically in R^91^. We compiled a GIS of data layers for each of the variables used in the paper.

In this paper we choose to analyze the centroids of geographic ranges rather than the entire geographic range for several reasons. First, not all the geographic ranges for all languages and mammal species are available. Second, it is not clear whether the ranges that are available are directly comparable. This is because the size of geographic ranges can be estimated many ways, and the distribution of species or languages within the geographic ranges can be complex. Population densities are not constant across ranges and the entire geographic range within any available boundary will not be sampled evenly. Therefore, having confidence in the complete geographic distribution of species or language speakers within their ranges (and the lacunae within them) requires additional levels of sampling not currently available. As such, while imperfect, we chose the centroid of the species and language geographic distributions as these metrics are more robust to measurement error while capturing the approximate central tendency of spatial diversity.

### Sampling procedure and statistics

Measuring diversity requires a sampling strategy. We use a statistical sampling technique designed to isolate the independent variable and reduce spatial autocorrelation. First, we divided the surface of the planet into a grid of 0.5 degrees of latitude by 0.5 degrees of longitude, extracted the non-oceanic cells (for a total of *n* = 56,598 cells) and calculated the available landmass per cell. For each cell we then counted the number of cultural populations and mammal species centroids that occur in that cell, and then divided that occurrence data by the available landmass. We also calculated the average temperature and net primary production for each cell. To sample global diversity, we then created bins of 0.1 lnNPP and 0.1 1/kT, and summed the total occurrence of ethnolinguistic and mammalian species densities, thus providing measures of the diversity per bin of independent variable controlling for the available landmass within that bin. This sampling approach has several important statistical advantages. First, we aggregate the data into bins by small increments of the independent variable, and then take statistics over the bin. This technique has the advantage of using the degrees of freedom available from the data set to estimate statistical moments within bins, and therefore isolates the effect of the independent variable on the dependent variable. Statistical error is thus minimized when estimating scaling exponents. Second, the binning procedure minimizes the effects of spatial autocorrelation as data points are aggregated from non-local populations collected from around the planet and averaged. Thus, data used in the scaling analyses are aggregates and are statistically independent. Third, the binning method minimizes the potential weighting biases of heteroscedasticity and unequal sampling across the data set.

Aside from the binning procedure, we do not attempt control for phylogenetic history in either of the datasets in this paper. This is because there is no phylogenetic supertree of human languages and so it is not statistically possible to control for the evolutionary structure of all languages in a single global database. Moreover, within the field of historical linguistics the concept of a supertree is highly controversial given that the speed at which languages evolve limit the ability to detect common ancestries beyond a few thousand years. Individual phylogenetic trees for most major language families of the world are now available, but not all, and some major geographic coverage is lacking, most notably for the Americas and Native American languages as a whole. While there is a phylogenetic supertree available for mammals we do not attempt to conduct phylogenetic comparisons on one database because this is not possible for the other. Therefore, comparing the two would be highly problematic and inconsistent. However, in the text we note that our binning procedure reshuffles data points distributed in space and therefore partially controls for the spatial autocorrelation inherent in evolutionary relationships, though we make no claim this is complete, nor equivalent to formal phylogenetic controls.

The data used in this paper are available in the Supplementary Material.

## Supplementary information

Supplementary information.

Supplementary information.

## References

[1] Mace R, Pagel M (1995). A latitudinal gradient in the density of human languages in North America. Proc. R. Soc. Lond. B.

[2] Nettle D (1999). Linguistic diversity of the Americas can be reconciled with a recent colonization. Proc. Natl. Acad. Sci..

[3] Collard IF, Foley RA (2002). Latitudinal patterns and environmental determinants of recent human cultural diversity: do humans follow biogeographical rules?. Evol. Ecol. Res..

[4] Pagel M, Mace R (2004). The cultural wealth of nations. Nature.

[5] Moore JL (2002). The distribution of cultural and biological diversity in Africa. Proc. R. Soc. Lond. B. Biol. Sci..

[6] Maffi L (2005). Linguistic, cultural, and biological diversity. Annu. Rev. Anthr..

[7] Harcourt A (2012). Human Biogeography.

[8] Willig MR, Kaufman DM, Stevens RD (2003). Latitudinal gradients of biodiversity: pattern, process, scale, and synthesis. Annu. Rev. Ecol. Evol. Syst..

[9] Rosenzweig ML (1995). Species Diversity in Space and Time.

[10] Pontarp M (2019). The latitudinal diversity gradient: novel understanding through mechanistic eco-evolutionary models. Trends Ecol. Evol..

[11] Rohde K (1992). Latitudinal gradients in species diversity: the search for the primary cause. Oikos.

[12] Belmaker J, Jetz W (2015). Relative roles of ecological and energetic constraints, diversification rates and region history on global species richness gradients. Ecol. Lett..

[13] Yasuhara M, Hunt G, Cronin TM, Okahashi H (2009). Temporal latitudinal-gradient dynamics and tropical instability of deep-sea species diversity. Proc. Natl. Acad. Sci..

[14] Kerkhoff AJ, Moriarty PE, Weiser MD (2014). The latitudinal species richness gradient in New World woody angiosperms is consistent with the tropical conservatism hypothesis. Proc. Natl. Acad. Sci..

[15] Stevens GC (1989). The latitudinal gradient in geographical range: how so many species coexist in the tropics. Am. Nat..

[16] Beech E, Rivers M, Oldfield S, Smith PP (2017). GlobalTreeSearch: the first complete global database of tree species and country distributions. J. Sustain. For..

[17] Roy K, Jablonski D, Martien KK (2000). Invariant size–frequency distributions along a latitudinal gradient in marine bivalves. Proc. Natl. Acad. Sci..

[18] Economo EP, Narula N, Friedman NR, Weiser MD, Guénard B (2018). Macroecology and macroevolution of the latitudinal diversity gradient in ants. Nat. Commun..

[19] Laenen B (2018). Evolutionary origin of the latitudinal diversity gradient in liverworts. Mol. Phylogenet. Evol..

[20] Guernier V, Hochberg ME, Guégan J-F (2004). Ecology drives the worldwide distribution of human diseases. PLoS Biol..

[21] Crame JA (2001). Taxonomic diversity gradients through geological time. Divers. Distrib..

[22] Mannion PD, Upchurch P, Benson RB, Goswami A (2014). The latitudinal biodiversity gradient through deep time. Trends Ecol. Evol..

[23] Nettle D (1998). Explaining global patterns of language diversity. J. Anthropol. Archaeol..

[24] Nettle D (1999). Linguistic Diversity.

[25] Allen AP, Brown JH, Gillooly JF (2002). Global biodiversity, biochemical kinetics, and the energetic-equivalence rule. Science.

[26] Brown JH (2014). Why are there so many species in the tropics?. J. Biogeogr..

[27] Hawkins BA (2003). Energy, water, and broad-scale geographic patterns of species richness. Ecology.

[28] Jablonski D (1993). The tropics as a source of evolutionary novelty through geological time. Nature.

[29] Savage VM (2004). Improved approximations to scaling relationships for species, populations, and ecosystems across latitudinal and elevational gradients. J. Theor. Biol..

[30] Nettle D (1996). Language diversity in West Africa: an ecological approach. J. Anthropol. Archaeol..

[31] Michaletz S, Cheng D, Kerkhoff A, Enquist B (2014). Convergence of terrestrial plant production across global climate gradients. Nature.

[32] Lomolino MV, Riddle BR, Whittaker RJ, Brown JH (2010). Biogeography.

[33] Brown JH (2014). Macroecology meets macroeconomics: resource scarcity and global sustainability. Ecol. Eng..

[34] Brown JH (2011). Energetic limits to economic growth. Bioscience.

[35] Nekola JC (2013). The Malthusian–Darwinian dynamic and the trajectory of civilization. Trends Ecol. Evol..

[36] Burger O, DeLong JP, Hamilton MJ (2011). Industrial energy use and the human life history. Sci. Rep..

[37] Burger JR, Weinberger VP, Marquet PA (2017). Extra-metabolic energy use and the rise in human hyper-density. Sci. Rep..

[38] Hutchinson GE (1959). Homage to Santa Rosalia or why are there so many kinds of animals?. Am. Nat..

[39] Brown JH (1981). Two decades of homage to Santa Rosalia: toward a general theory of diversity. Am. Zool..

[40] Gavin MC (2017). Process-based modelling shows how climate and demography shape language diversity. Glob. Ecol. Biogeogr..

[41] Derungs C, Köhl M, Weibel R, Bickel B (2018). Environmental factors drive language density more in food-producing than in hunter–gatherer populations. Proc. R. Soc. B Biol. Sci..

[42] Gavin MC (2013). Toward a mechanistic understanding of linguistic diversity. Bioscience.

[43] Túlio PCM (2019). Drivers of geographical patterns of North American language diversity. Proc. R. Soc. B Biol. Sci..

[44] Tallavaara M, Eronen JT, Luoto M (2018). Productivity, biodiversity, and pathogens influence the global hunter-gatherer population density. Proc. Natl. Acad. Sci..

[45] Currie TE, Mace R (2009). Political complexity predicts the spread of ethnolinguistic groups. Proc. Natl. Acad. Sci..

[46] Hamilton MJ, Milne BT, Walker RS, Brown JH (2007). Nonlinear scaling of space use in human hunter–gatherers. Proc. Natl. Acad. Sci..

[47] Hamilton MJ, Lobo J, Rupley E, Youn H, West GB (2016). The ecological and evolutionary energetics of hunter-gatherer residential mobility. Evol. Anthropol. Issues News Rev..

[48] Hamilton MJ, Walker RS, Buchanan B, Sandeford DS (2020). Scaling human sociopolitical complexity. PLoS ONE.

[49] Enquist BJ (2003). Scaling metabolism from organisms to ecosystems. Nature.

[50] Kleiber, M. The fire of life. An introduction to animal energetics. *Fire Life Introd. Anim. Energ.* (1961).

[51] Brummer AB, Savage VM, Enquist BJ (2017). A general model for metabolic scaling in self-similar asymmetric networks. PLoS Comput. Biol..

[52] Hulbert AJ (2014). A sceptics view: “Kleiber’s Law” or the “3/4 Rule” is neither a law nor a rule but rather an empirical approximation. Systems.

[53] Ballesteros FJ (2018). On the thermodynamic origin of metabolic scaling. Sci. Rep..

[54] Kolokotrones T, Savage V, Deeds EJ, Fontana W (2010). Curvature in metabolic scaling. Nature.

[55] West GB, Brown JH, Enquist BJ (1997). A general model for the origin of allometric scaling laws in biology. Science.

[56] West GB, Brown JH, Enquist BJ (1999). The fourth dimension of life: fractal geometry and allometric scaling of organisms. Science.

[57] Brown JH, Gillooly JF, Allen AP, Savage VM, West GB (2004). Toward a metabolic theory of ecology. Ecology.

[58] Savage VM (2004). The predominance of quarter-power scaling in biology. Funct. Ecol..

[59] Hunt D, Savage VM (2016). Asymmetries arising from the space-filling nature of vascular networks. Phys. Rev. E.

[60] Gillooly JF, Brown JH, West GB, Savage VM, Charnov EL (2001). Effects of size and temperature on metabolic rate. Science.

[61] Brown JH, Sibly RM, Sibly RM, Brown JH, Kodric-Brown A (2012). The metabolic theory of ecology and its central equation. Metabolic Ecology: A Scaling Approach.

[62] Anderson-Teixeira KJ, Vitousek PM, Sibly RM, Brown JH, Kodric-Brown A (2012). Ecosystems. Metabolic Ecology: A Scaling Approach.

[63] Chapin FS, Matson PA, Vitousek P (2011). Principles of Terrestrial Ecosystem Ecology.

[64] Falkowski P (2000). The global carbon cycle: a test of our knowledge of earth as a system. Science.

[65] Williams M (1997). Predicting gross primary productivity in terrestrial ecosystems. Ecol. Appl..

[66] Allen AP, Gillooly JF, Brown JH (2005). Linking the global carbon cycle to individual metabolism. Funct. Ecol..

[67] Anderson KJ, Allen AP, Gillooly JF, Brown JH (2006). Temperature-dependence of biomass accumulation rates during secondary succession. Ecol. Lett..

[68] Gillman LN, Keeling DJ, Gardner RC, Wright SD (2010). Faster evolution of highly conserved DNA in tropical plants. J. Evol. Biol..

[69] Mittelbach GG (2007). Evolution and the latitudinal diversity gradient: speciation, extinction and biogeography. Ecol. Lett..

[70] Martínez-Meyer E, Townsend Peterson A, Hargrove WW (2004). Ecological niches as stable distributional constraints on mammal species, with implications for Pleistocene extinctions and climate change projections for biodiversity. Glob. Ecol. Biogeogr..

[71] Hubbell SP (2001). The Unified Neutral Theory of Biodiversity and Biogeography (MPB-32).

[72] MacArthur RH (1984). Geographical Ecology: Patterns in the Distribution of Species.

[73] Richerson PJ, Boyd R (2005). Not By Genes Alone.

[74] Henrich J (2017). The Secret of Our Success: How Culture is Driving Human Evolution, Domesticating Our Species, and Making Us Smarter.

[75] Turchin, P. *Ultrasociety: How 10,000 Years of War Made Humans the Greatest Cooperators on Earth*. (Beresta Books, 2015).

[76] Van Valen L (1977). The red queen. Am. Nat..

[77] Perreault C (2012). The Pace of cultural evolution. PLoS ONE.

[78] Perreault C (2019). The Quality of the Archaeological Record.

[79] Greenhill SJ, Atkinson QD, Meade A, Gray RD (2010). The shape and tempo of language evolution. Proc. R. Soc. Lond. B Biol. Sci..

[80] Moore, G. E. Cramming more components onto integrated circuits, Electronics, 38: 8 (1965). *URL Ftpdownload Intel Comresearchsiliconmoorespaper Pdf* **16**, (2005).

[81] Youn H, Strumsky D, Bettencourt LM, Lobo J (2015). Invention as a combinatorial process: evidence from US patents. J. R. Soc. Interface.

[82] Magurran AE (2004). Measuring Biological Diversity.

[83] Cavalli-Sforza LL, Feldman MW (1981). Cultural Transmission and Evolution: A Quantitative Approach.

[84] Henrich J, McElreath R (2003). The evolution of cultural evolution. Evol. Anthropol. Issues News Rev..

[85] Prothero DR (2014). Species longevity in North American fossil mammals. Integr. Zool..

[86] Erwin DH (2000). Macroevolution is more than repeated rounds of microevolution. Evol. Dev..

[87] Walker RS, Wichmann S, Mailund T, Atkisson CJ (2012). Cultural phylogenetics of the Tupi language family in lowland South America. PLoS ONE.

[88] Hijmans RJ, Cameron SE, Parra JL, Jones PG, Jarvis A (2005). Very high resolution interpolated climate surfaces for global land areas. Int. J. Climatol..

[89] Cramer W (1999). Comparing global models of terrestrial net primary productivity (NPP): overview and key results. Glob. Change Biol..

[90] Wessel P, Smith WH (1996). A global, self-consistent, hierarchical, high-resolution shoreline database. J. Geophys. Res. Solid Earth.

[91] Team, R. C. *R: A Language and Environment for Statistical Computing. R Foundation for Statistical Computing, Vienna, Austria. 2013*. (2014).

