# Supplementary material for: Diversity begets diversity in mammal species and human cultures
Source: Sci Rep. 2020 Nov 12;10:19654. doi: 10.1038/s41598-020-76658-2 (PMC7661729; doi:10.1038/s41598-020-76658-2)
Supplement: Supplementary file 1 — Supplementary information. [file 41598_2020_76658_MOESM1_ESM.docx]

Supplementary Information for “Diversity begets diversity in mammal species and human cultures”

Marcus J. Hamilton^1,2,^*

Robert S. Walker^3^

Christopher P. Kempes^2^

^1^ Department of Anthropology, University of Texas at San Antonio, San Antonio, TX

^2^ Santa Fe Institute, Santa Fe, NM

^3^ Department of Anthropology, University of Missouri, Columbia, MO

* Corresponding author

**Detailed statistical Results:**

The following are details of each statistical model performed in the paper presented in the sequence they occur in the paper, and labeled by the figures in the main text. The data used to perform these models is available in the second file of the Supplementary Information.

Figure 3A.

**Regression Analysis: Mammal diversity and latitude (lnMammal dens versus abslat)**

Method

| Rows unused | 46 |
| --- | --- |

Analysis of Variance

| Source | DF | Seq SS | Contribution | Adj SS | Adj MS | F-Value | P-Value |
| --- | --- | --- | --- | --- | --- | --- | --- |
| Regression | 1 | 66.12 | 75.88% | 66.12 | 66.1158 | 207.66 | 0.000 |
| abslat | 1 | 66.12 | 75.88% | 66.12 | 66.1158 | 207.66 | 0.000 |
| Error | 66 | 21.01 | 24.12% | 21.01 | 0.3184 |  |  |
| Total | 67 | 87.13 | 100.00% |  |  |  |  |

Model Summary

| S | R-sq | R-sq(adj) | PRESS | R-sq(pred) |
| --- | --- | --- | --- | --- |
| 0.564249 | 75.88% | 75.52% | 22.5431 | 74.13% |

Coefficients

| Term | Coef | SE Coef | 95% CI | T-Value | P-Value | VIF |
| --- | --- | --- | --- | --- | --- | --- |
| Constant | 4.333 | 0.134 | (4.065, 4.601) | 32.31 | 0.000 |  |
| abslat | -0.04926 | 0.00342 | (-0.05609, -0.04244) | -14.41 | 0.000 | 1.00 |

Regression Equation

| lnMammal dens | = | 4.333 - 0.04926 abslat |
| --- | --- | --- |

**Regression Analysis: Ethnolinguistic diversity and latitude ( lnEthno dens versus abslat)**

Method

| Rows unused | 8 |
| --- | --- |

Analysis of Variance

| Source | DF | Seq SS | Contribution | Adj SS | Adj MS | F-Value | P-Value |
| --- | --- | --- | --- | --- | --- | --- | --- |
| Regression | 1 | 76.48 | 82.53% | 76.48 | 76.4799 | 335.43 | 0.000 |
| abslat | 1 | 76.48 | 82.53% | 76.48 | 76.4799 | 335.43 | 0.000 |
| Error | 71 | 16.19 | 17.47% | 16.19 | 0.2280 |  |  |
| Total | 72 | 92.67 | 100.00% |  |  |  |  |

Model Summary

| S | R-sq | R-sq(adj) | PRESS | R-sq(pred) |
| --- | --- | --- | --- | --- |
| 0.477496 | 82.53% | 82.28% | 17.3409 | 81.29% |

Coefficients

| Term | Coef | SE Coef | 95% CI | T-Value | P-Value | VIF |
| --- | --- | --- | --- | --- | --- | --- |
| Constant | 4.915 | 0.111 | (4.694, 5.135) | 44.45 | 0.000 |  |
| abslat | -0.04852 | 0.00265 | (-0.05380, -0.04324) | -18.31 | 0.000 | 1.00 |

Regression Equation

| lnEthno dens | = | 4.915 - 0.04852 abslat |
| --- | --- | --- |

Figure 3B.

**Regression Analysis: Mammal diversity and temperature ( lnM/A versus 1/kT)**

Method

| Rows unused | 28 |
| --- | --- |

Analysis of Variance

| Source | DF | Seq SS | Contribution | Adj SS | Adj MS | F-Value | P-Value |
| --- | --- | --- | --- | --- | --- | --- | --- |
| Regression | 1 | 97.87 | 86.81% | 97.87 | 97.8703 | 388.33 | 0.000 |
| 1/kT | 1 | 97.87 | 86.81% | 97.87 | 97.8703 | 388.33 | 0.000 |
| Error | 59 | 14.87 | 13.19% | 14.87 | 0.2520 |  |  |
| Total | 60 | 112.74 | 100.00% |  |  |  |  |

Model Summary

| S | R-sq | R-sq(adj) | PRESS | R-sq(pred) |
| --- | --- | --- | --- | --- |
| 0.502023 | 86.81% | 86.59% | 16.2772 | 85.56% |

Coefficients

| Term | Coef | SE Coef | 95% CI | T-Value | P-Value | VIF |
| --- | --- | --- | --- | --- | --- | --- |
| Constant | 31.82 | 1.50 | (28.81, 34.82) | 21.21 | 0.000 |  |
| 1/kT | -0.7080 | 0.0359 | (-0.7799, -0.6361) | -19.71 | 0.000 | 1.00 |

Regression Equation

| lnM/A | = | 31.82 - 0.7080 1/kT |
| --- | --- | --- |

**Regression Analysis: Ethnolinguistic diversity and temperature (lnE/A versus 1/kT)**

Method

| Rows unused | 23 |
| --- | --- |

Analysis of Variance

| Source | DF | Seq SS | Contribution | Adj SS | Adj MS | F-Value | P-Value |
| --- | --- | --- | --- | --- | --- | --- | --- |
| Regression | 1 | 120.86 | 90.57% | 120.86 | 120.856 | 614.83 | 0.000 |
| 1/kT | 1 | 120.86 | 90.57% | 120.86 | 120.856 | 614.83 | 0.000 |
| Error | 64 | 12.58 | 9.43% | 12.58 | 0.197 |  |  |
| Total | 65 | 133.44 | 100.00% |  |  |  |  |

Model Summary

| S | R-sq | R-sq(adj) | PRESS | R-sq(pred) |
| --- | --- | --- | --- | --- |
| 0.443361 | 90.57% | 90.42% | 13.5080 | 89.88% |

Coefficients

| Term | Coef | SE Coef | 95% CI | T-Value | P-Value | VIF |
| --- | --- | --- | --- | --- | --- | --- |
| Constant | 32.09 | 1.19 | (29.72, 34.47) | 27.00 | 0.000 |  |
| 1/kT | -0.7016 | 0.0283 | (-0.7581, -0.6451) | -24.80 | 0.000 | 1.00 |

Regression Equation

| lnE/A | = | 32.09 - 0.7016 1/kT |
| --- | --- | --- |

Figure 4.

**Regression Analysis: Mammal diversity and net primary production (lnM/A versus lnNPP)**

Method

| Rows unused | 18 |
| --- | --- |

Analysis of Variance

| Source | DF | Seq SS | Contribution | Adj SS | Adj MS | F-Value | P-Value |
| --- | --- | --- | --- | --- | --- | --- | --- |
| Regression | 1 | 88.603 | 91.23% | 88.603 | 88.6033 | 395.41 | 0.000 |
| lnNPP | 1 | 88.603 | 91.23% | 88.603 | 88.6033 | 395.41 | 0.000 |
| Error | 38 | 8.515 | 8.77% | 8.515 | 0.2241 |  |  |
| Total | 39 | 97.118 | 100.00% |  |  |  |  |

Model Summary

| S | R-sq | R-sq(adj) | PRESS | R-sq(pred) |
| --- | --- | --- | --- | --- |
| 0.473369 | 91.23% | 91.00% | 9.75006 | 89.96% |

Coefficients

| Term | Coef | SE Coef | 95% CI | T-Value | P-Value | VIF |
| --- | --- | --- | --- | --- | --- | --- |
| Constant | -3.512 | 0.325 | (-4.170, -2.854) | -10.81 | 0.000 |  |
| lnNPP | 1.2279 | 0.0617 | (1.1029, 1.3529) | 19.88 | 0.000 | 1.00 |

Regression Equation

| lnM/A | = | -3.512 + 1.2279 lnNPP |
| --- | --- | --- |

**Regression Analysis: Ethnolinguistic diversity and net primary production lnE/A versus lnNPP**

Method

| Rows unused | 19 |
| --- | --- |

Analysis of Variance

| Source | DF | Seq SS | Contribution | Adj SS | Adj MS | F-Value | P-Value |
| --- | --- | --- | --- | --- | --- | --- | --- |
| Regression | 1 | 99.64 | 90.77% | 99.64 | 99.6408 | 363.89 | 0.000 |
| lnNPP | 1 | 99.64 | 90.77% | 99.64 | 99.6408 | 363.89 | 0.000 |
| Error | 37 | 10.13 | 9.23% | 10.13 | 0.2738 |  |  |
| Total | 38 | 109.77 | 100.00% |  |  |  |  |

Model Summary

| S | R-sq | R-sq(adj) | PRESS | R-sq(pred) |
| --- | --- | --- | --- | --- |
| 0.523280 | 90.77% | 90.52% | 12.1006 | 88.98% |

Coefficients

| Term | Coef | SE Coef | 95% CI | T-Value | P-Value | VIF |
| --- | --- | --- | --- | --- | --- | --- |
| Constant | -3.994 | 0.389 | (-4.782, -3.205) | -10.26 | 0.000 |  |
| lnNPP | 1.3967 | 0.0732 | (1.2484, 1.5451) | 19.08 | 0.000 | 1.00 |

Regression Equation

| lnE/A | = | -3.994 + 1.3967 lnNPP |
| --- | --- | --- |

Figure 5A.

**Regression Analysis: Mammal diversity and temperature in bins of net primary production (lnM/A versus 1/kT, lnNPPbin)**

Method

| Categorical predictor coding | (1, 0) |
| --- | --- |
| Rows unused | 411 |

Analysis of Variance

| Source | DF | Seq SS | Contribution | Adj SS | Adj MS | F-Value | P-Value |
| --- | --- | --- | --- | --- | --- | --- | --- |
| Regression | 7 | 240.84 | 64.40% | 240.836 | 34.4051 | 52.71 | 0.000 |
| 1/kT | 1 | 100.71 | 26.93% | 4.504 | 4.5041 | 6.90 | 0.009 |
| lnNPPbin | 3 | 100.26 | 26.81% | 42.309 | 14.1029 | 21.61 | 0.000 |
| 1/kT*lnNPPbin | 3 | 39.87 | 10.66% | 39.867 | 13.2890 | 20.36 | 0.000 |
| Error | 204 | 133.16 | 35.60% | 133.162 | 0.6528 |  |  |
| Total | 211 | 374.00 | 100.00% |  |  |  |  |

Model Summary

| S | R-sq | R-sq(adj) | PRESS | R-sq(pred) |
| --- | --- | --- | --- | --- |
| 0.807931 | 64.40% | 63.17% | 148.123 | 60.39% |

Coefficients

| Term | Coef | SE Coef | 95% CI | T-Value | P-Value | VIF |
| --- | --- | --- | --- | --- | --- | --- |
| Constant | 1.29 | 3.16 | (-4.94, 7.52) | 0.41 | 0.683 |  |
| 1/kT | -0.2035 | 0.0775 | (-0.3563, -0.0508) | -2.63 | 0.009 | 6.02 |
| lnNPPbin |  |  |  |  |  |  |
| 4 | 0.000000 | 0.000000 | (0.000000, 0.000000) | * | * | * |
| 5 | 5.08 | 3.92 | (-2.65, 12.81) | 1.30 | 0.196 | 1022.69 |
| 6 | 7.53 | 3.94 | (-0.24, 15.29) | 1.91 | 0.057 | 1042.28 |
| 7 | 31.12 | 4.36 | (22.51, 39.72) | 7.13 | 0.000 | 1083.51 |
| 1/kT*lnNPPbin |  |  |  |  |  |  |
| 4 | 0.000000 | 0.000000 | (0.000000, 0.000000) | * | * | * |
| 5 | -0.1000 | 0.0956 | (-0.2886, 0.0885) | -1.05 | 0.297 | 1043.96 |
| 6 | -0.1388 | 0.0961 | (-0.3284, 0.0507) | -1.44 | 0.150 | 1060.36 |
| 7 | -0.723 | 0.107 | (-0.934, -0.512) | -6.76 | 0.000 | 1084.28 |

Regression Equation

| lnNPPbin |  |  |  |
| --- | --- | --- | --- |
| 4 | lnM/A | = | 1.29 - 0.2035 1/kT |
| 5 | lnM/A | = | 6.37 - 0.3036 1/kT |
| 6 | lnM/A | = | 8.82 - 0.3424 1/kT |
| 7 | lnM/A | = | 32.41 - 0.9267 1/kT |

Figure 5B.

**Regression Analysis: Ethnolinguistic diversity and temperature in bins of net primary production (lnE/A versus 1/kT, lnNPPbin)**

Method

| Categorical predictor coding | (1, 0) |
| --- | --- |
| Rows unused | 405 |

Analysis of Variance

| Source | DF | Seq SS | Contribution | Adj SS | Adj MS | F-Value | P-Value |
| --- | --- | --- | --- | --- | --- | --- | --- |
| Regression | 7 | 340.46 | 70.58% | 340.46 | 48.6371 | 71.97 | 0.000 |
| 1/kT | 1 | 142.37 | 29.52% | 12.99 | 12.9926 | 19.23 | 0.000 |
| lnNPPbin | 3 | 167.95 | 34.82% | 33.82 | 11.2735 | 16.68 | 0.000 |
| 1/kT*lnNPPbin | 3 | 30.13 | 6.25% | 30.13 | 10.0449 | 14.86 | 0.000 |
| Error | 210 | 141.91 | 29.42% | 141.91 | 0.6758 |  |  |
| Total | 217 | 482.37 | 100.00% |  |  |  |  |

Model Summary

| S | R-sq | R-sq(adj) | PRESS | R-sq(pred) |
| --- | --- | --- | --- | --- |
| 0.822055 | 70.58% | 69.60% | 156.006 | 67.66% |

Coefficients

| Term | Coef | SE Coef | 95% CI | T-Value | P-Value | VIF |
| --- | --- | --- | --- | --- | --- | --- |
| Constant | 7.39 | 3.22 | (1.03, 13.74) | 2.29 | 0.023 |  |
| 1/kT | -0.3479 | 0.0793 | (-0.5043, -0.1915) | -4.38 | 0.000 | 7.27 |
| lnNPPbin |  |  |  |  |  |  |
| 4 | 0.000000 | 0.000000 | (0.000000, 0.000000) | * | * | * |
| 5 | -1.04 | 3.93 | (-8.79, 6.70) | -0.27 | 0.791 | 1013.45 |
| 6 | 8.10 | 3.86 | (0.49, 15.71) | 2.10 | 0.037 | 1023.31 |
| 7 | 22.72 | 4.25 | (14.34, 31.09) | 5.35 | 0.000 | 1085.73 |
| 1/kT*lnNPPbin |  |  |  |  |  |  |
| 4 | 0.000000 | 0.000000 | (0.000000, 0.000000) | * | * | * |
| 5 | 0.0467 | 0.0960 | (-0.1426, 0.2360) | 0.49 | 0.627 | 1044.27 |
| 6 | -0.1383 | 0.0943 | (-0.3242, 0.0476) | -1.47 | 0.144 | 1061.00 |
| 7 | -0.508 | 0.104 | (-0.713, -0.302) | -4.87 | 0.000 | 1095.73 |

Regression Equation

| lnNPPbin |  |  |  |
| --- | --- | --- | --- |
| 4 | lnE/A | = | 7.39 - 0.3479 1/kT |
| 5 | lnE/A | = | 6.35 - 0.3012 1/kT |
| 6 | lnE/A | = | 15.49 - 0.4862 1/kT |
| 7 | lnE/A | = | 30.10 - 0.8557 1/kT |
